# Effects of Nitrogen Oxides on the Production of Reactive Oxygen Species and Environmentally Persistent Free Radicals from α-Pinene and Naphthalene Secondary Organic Aerosols

**DOI:** 10.1021/acs.jpca.2c05532

**Published:** 2022-10-04

**Authors:** Kasey C. Edwards, Alexandra L. Klodt, Tommaso Galeazzo, Meredith Schervish, Jinlai Wei, Ting Fang, Neil M. Donahue, Bernard Aumont, Sergey A. Nizkorodov, Manabu Shiraiwa

**Affiliations:** † Department of Chemistry, University of California, Irvine, Irvine, California 92697, United States; ‡ Departments of Chemistry, Chemical Engineering, Engineering and Public Policy, Center for Atmospheric Particle Studies, Carnegie Mellon University, Pittsburgh, Pennsylvania 15213, United States; § CNRS, LISA, Univ of Paris Est Creteil and University Paris Cité, F-94010 Créteil, France

## Abstract

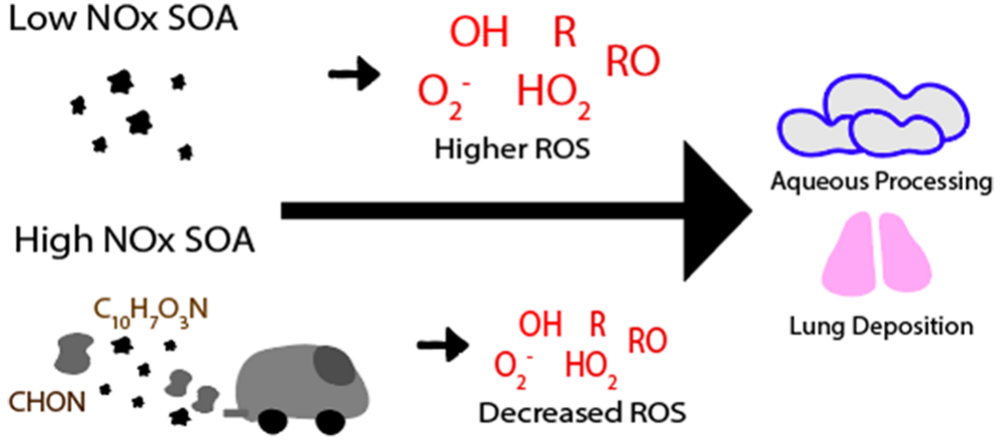

Reactive oxygen species (ROS) and environmentally persistent free radicals (EPFR) play an important role in chemical transformation of atmospheric aerosols and adverse aerosol health effects. This study investigated the effects of nitrogen oxides (NO_*x*_) during photooxidation of α-pinene and naphthalene on the EPFR content and ROS formation from secondary organic aerosols (SOA). Electron paramagnetic resonance (EPR) spectroscopy was applied to quantify EPFR content and ROS formation. While no EPFR were detected in α-pinene SOA, we found that naphthalene SOA contained about 0.7 pmol μg^–1^ of EPFR, and NO_*x*_ has little influence on EPFR concentrations and oxidative potential. α-Pinene and naphthalene SOA generated under low NO_*x*_ conditions form OH radicals and superoxide in the aqueous phase, which was lowered substantially by 50–80% for SOA generated under high NO_*x*_ conditions. High-resolution mass spectrometry analysis showed the substantial formation of nitroaromatics and organic nitrates in a high NO_*x*_ environment. The modeling results using the GECKO-A model that simulates explicit gas-phase chemistry and the radical 2D-VBS model that treats autoxidation predicted reduced formation of hydroperoxides and enhanced formation of organic nitrates under high NO_*x*_ due to the reactions of peroxy radicals with NO_*x*_ instead of their reactions with HO_2_. Consistently, the presence of NO_*x*_ resulted in the decrease of peroxide contents and oxidative potential of α-pinene SOA.

## Introduction

Atmospheric aerosols play an important role in climate, atmospheric chemistry, and public health.^1−5^ Organic aerosols account for 20–90% of the total aerosol mass in the troposphere.^6,7^ They can either be directly emitted into the atmosphere or be generated through oxidation of volatile organic compounds (VOC) by ozone (O_3_), hydroxyl radicals (OH), and nitrate radicals (NO_3_), followed by nucleation or condensation of semivolatile and low volatility products to form secondary organic aerosols (SOA).^8,9^ The chemical composition of SOA can vary widely based on the environments in which they are formed. It has been well studied that the presence of nitrogen oxides (NO_*x*_ = NO + NO_2_), emitted mainly from vehicular exhaust in urban air, can modulate gas-phase chemistry and chemical composition of SOA significantly.^10−14^

The OH oxidation of VOC is initiated by either abstraction of hydrogen or addition of OH to a C=C double bond, followed by the addition of molecular oxygen to generate peroxy radicals (RO_2_). Peroxy radicals have a number of reaction pathways with hydroperoxy radicals (HO_2_), nitrogen monoxide (NO), nitrogen dioxide (NO_2_), and other peroxy radicals (RO_2_). They can also undergo isomerization by internal hydrogen shift followed by molecular oxygen addition, so-called autoxidation.^15,16^ The fate of RO_2_ and reaction products is strongly affected by the level of NO_*x*_: organic hydroperoxides (ROOH) are major reaction products as generated by reactions with HO_2_ or autoxidation to form highly oxygenated organic molecules (HOM) under low NO_*x*_,^17^ while organic nitrates as well as carbonyls are expected to form by reactions with NO_*x*_ under high NO_*x*_.

Reactive oxygen species (ROS) including hydrogen peroxide (H_2_O_2_), superoxide (O_2_^•–^), hydroxyl radical (OH), hydroperoxy radical (HO_2_), and organic radicals are contained within aerosols, triggering chemical transformation of aerosols in the atmosphere and oxidative stress upon respiratory deposition. There are growing numbers of studies to measure oxidative potential of particulate matter,^18^ which may represent the redox activity for generation of ROS.^19,20^ Previous studies have shown that ROS can be generated by aqueous reactions of SOA components such as decomposition of ROOH to generate OH followed by a cascade of aqueous reactions involving alcohols to generate superoxide.^21,22^ Inhalation and respiratory deposition of SOA can lead to ROS generation in lung lining fluid, and excess ROS may cause oxidative damage and stress, inflammation, biological aging, and cell death.^23−26^ Previous research indicated that aqueous-phase OH production is reduced in the presence of NO_2_.^27^ A study by Chowdhury et al., which generated SOA under low and high NO_*x*_ conditions in an oxidative flow reactor with ∼3 days of equivalent atmospheric aging, reported that NO_*x*_ had no effect on ROS or total peroxide contents for naphthalene SOA, while NO_*x*_ increased ROS generation with no effect on total peroxide content in α-pinene SOA.^28^ Our study follows up these studies by investigating ROS formation from chamber-generated SOA with atmospheric aging on the hour scale.

In addition to production of ROS, which are relatively short-lived due to their highly reactive nature, SOA derived from aromatic precursors are observed to contain stable and long-lived radicals, so-called environmentally persistent free radicals (EPFR).^29,30^ EPFR have longer lifetimes from minutes, months, and even indefinite in the atmosphere.^31−34^ Naphthalene and other polycyclic aromatic compounds can produce EPFR upon oxidation to form semiquinone radicals, which are redox-active to produce ROS.^27,35^ Only a handful of studies have investigated the effects of NO_*x*_ on EPFR production. Gehling and Dellinger suggested that NO_2_ has the potential to decrease ROS production from EPFR in ambient air, while NO has little to no effect.^27^

Electron paramagnetic resonance (EPR) spectroscopy is an analytical technique that can be used to directly measure radicals.^10,36^ In this study, we investigate ROS formation by SOA generated from representative anthropogenic and biogenic VOC precursors, naphthalene and α-pinene, respectively, under low and high NO_*x*_ conditions to evaluate the effects of NO_*x*_ on EPFR and ROS formation from SOA through EPR analysis. In addition, high-resolution mass spectrometry was used to determine chemical composition of SOA generated in high and low NO_*x*_ environments. We also simulated the chemical composition of α-pinene SOA using an explicit gas-phase chemistry model (GECKO-A) and the radical two-dimensional volatility basis set (r2D-VBS) to estimate the distribution of functional groups in SOA.

## Materials and Methods

### Secondary Organic Aerosol Generation

SOA particles were generated from the photooxidation of naphthalene or α-pinene with or without the presence of NO_*x*_ in an environmental chamber. The chamber consists of a 5 m^3^ Teflon bag surrounded by a bank of 42 UV-B lamps with an emission spectrum centered at 310 nm, equipped with a scanning mobility particle sizer (SMPS) (TSI model 3936 classifier and model 3775 condensation particle counter) to monitor particle size distributions as well as an ozone monitor (Thermo Scientific model 49i) and an NO_*y*_ monitor (Thermo Scientific Model 42i-Y). SMPS, NO_*y*_, and ozone data are available online at the Index of Chamber Atmospheric Research in the United States (ICARUS).^37^ Before the injection of VOC and oxidant, the chamber was humidified to about 40%. The experiments were conducted at ambient temperature, which was 21 ± 2 °C for the duration of all experiments, with most of the variation being from heating by the lamps, which also reduced the relative humidity (RH) by several percentage points. Experiments were performed by injecting 2000 ppb of H_2_O_2_ as the OH precursor followed by 200 ppb of naphthalene or 500 ppb of α-pinene through a heated inlet. The steady-state OH concentration was estimated to be ∼1.4 × 10^6^ molecules cm^–3^, similar to previous work.^38^ For the high NO_*x*_ experiments, 700 ppb of NO was added. No additional VOC, H_2_O_2_, or NO was added after the start of the experiment. Photooxidation was performed for 3.0 ± 0.5 h for all experiments for consistency. SOA particles were then collected on a 0.2 μm PTFE filter (Merck Millipore Ltd., part number FGLP04700) at a flow rate of 20 L min^–1^. The mass difference of the filter before and after collection was used as the SOA collection mass for all mass corrected results. A molar mass of 200 g mol^–1^ was assumed for all SOA collected and used to calculate yield.^39^

### EPFR and ROS

After sample collection, the filters were immediately analyzed for EPFR using a X-band continuous-wave electron paramagnetic resonance (EPR) spectrometer (Bruker, Germany). The EPFR concentrations were quantified using a calibration curve of 4-hydroxy-2,2,6,6-tetramethylpiperidine-1-oxyl (TEMPOL). EPFR measurements were scanned over the field range of 3300–3700 G and recorded as an average of 10 scans. The parameters for EPFR measurements were as follows: an attenuation of 12 dB, a modulation amplitude of 1.0 G, a microwave power 12.62 mW, and a receiver gain of 40. For EPFR analysis the filter was placed into a quartz tube and then directly placed in the resonator. EPFR stability was tested by analyzing filters at 5–10 min intervals up to 1.5 h postcollection. The filter was then stored at −20 °C until used for ROS analysis. For ROS measurements, the filter was extracted in a 1 mL aqueous solution of 10 mM spin-trapping agent 5-*tert*-butoxycarbonyl-5-methyl-1-pyrroline-*N*-oxide (BMPO) (Enzo, >99%). The extract was then placed in a 50 μL capillary tube for EPR measurements. The samples were scanned over the field range of 3470–3560 G and averaged over 50 scans. The parameters for ROS measurements were the same as EPFR, except for the receiver gain set to 30. The Bruker SpinFit software was used to deconvolute the EPR spectra to quantify the concentrations of BMPO adducts with hydroxyl radical (OH), superoxide (O_2_^•–^)/hydroperoxyl radical (HO_2_), and carbon- and oxygen-centered organic radicals.^33,34^

### Total Peroxide

A second set of chamber filters was generated for a total peroxide measurement and dithiothreitol (DTT) analysis. Peroxides can oxidize I^–^ to form I_2_, which can then combine with I^–^ to form I_3_^–^. Peroxides in solution can be quantified by the characteristic absorbance of I_3_^–^ at 289 and 350 nm. A wavelength of 350 nm was used to quantify peroxide concentration in naphthalene and α-pinene SOA. Calibration was performed using 0.2–2 μM benzoyl peroxide (Sigma-Aldrich, ≥98%). SOA filters were extracted for 7 min in 1 mL of Milli-Q water. 100 μL of the SOA extract was combined with 700 μL of ethyl acetate (Sigma-Aldrich, 99.8%). The 800 μL solution was then mixed with 636 μL of acetic acid (Sigma-Aldrich, ≥99%), 324 μL of chloroform (Sigma-Aldrich, ≥99.5%), and 240 μL of Milli-Q water, forming a 2 mL solution. This solution was then purged of dissolved O_2_, which might also oxidize I^–^, by a N_2_ flow for 2 min. Afterward, 20 mg of potassium iodide (KI, Sigma-Aldrich, ≥99%) was added to the solution, and it was left to sit for 1 h. The absorbance was then measured at 405 nm using the GloMax Discover Microplate Reader. The total peroxide measurement method is based on the iodometric–spectrophotometric method used by Docherty et al.^40,41^

### DTT Assay

Dithiothreitol (DTT) analysis was performed to measure the total oxidative potential and redox activity,^32,42^ which is often assumed to correspond to ROS formation and to represent indicator of PM toxicity.^20^ DTT analysis quantifies the consumption of DTT over time by redox-active or reactive compounds contained in SOA water extracts. The sample was extracted into 0.7 mL of Milli-Q water and combined with 0.2 mL of potassium phosphate buffer (pH 7.4) in the reaction vial. The vial was then incubated at 37 °C, and the reaction was initiated by adding 0.1 mL of 1 mM DTT. A 50 μL aliquot of the reaction vial was then mixed with trichloroacetic acid (TCA) to quench the reaction and different time points to note the oxidation over time. To analyze the sample, it was mixed with Tris buffer and 5,5-dithiobis(2-nitrobenzoic acid) (DTNB), which combines with the residual DTT to form a light-absorbing compound. The absorbance of this product was measured at 412 nm with the Liquid Waveguide Capillary Cell (LWCC) coupled to an online spectrophotometer (Ocean Optics, Inc., Dunedin, FL), consisting of an DT-Mini-2 ultraviolet– visible (UV–vis) light source and a USB4000 miniature fiber-optic spectrometer.

### High-Resolution Mass Spectrometry (HRMS)

HRMS was used to analyze SOA chemical composition. SOA was extracted from the filters by shaking the filters in acetonitrile for 5 min. The solvent volume was chosen to achieve a concentration of SOA in acetonitrile of 400 μg mL^–1^ assuming 100% extraction efficiency. Then an equal amount of water was added so that the SOA concentration would be 200 μg mL^–1^ for HRMS analysis.

The instrument has been described previously in Chin et al.^43^ A 10 μL aliquot of sample was injected into a Phenomenex Luna Omega Polar C18, 150 × 2.1 mm^2^ column, with 1.6 μm particles and 100 Å pores for ultrahigh performance liquid chromatography (UPLC) separation and photodiode array (PDA) detection. The PDA detector was followed by a Thermo Q-Exactive Plus mass spectrometer with a heated electrospray ionization inlet and a resolving power of 1.4 × 10^5^ at *m*/*z* 400, which was operated in both positive (spray voltage +3.5 kV) and negative (spray voltage −2.5 kV) ion modes.^43^ The UPLC solvents were water acidified to pH 3 with 0.1 wt % formic acid (solvent A) and acetonitrile acidified with 0.1 wt % formic acid (solvent B). The gradient was 95% solvent A and 5% solvent B for 3 min, followed by a linear ramp to 95% solvent B and 5% solvent A from for 11 min, a hold at 95% solvent B for 2 min, and finally a linear ramp back to 95% solvent A and 5% solvent B for 4 min in preparation for the next run.

FreeStyle 1.6 from Thermo Scientific was used to generate a raw time-integrated mass spectrum by integrating over the full total ion chromatogram (1–18 min). Peaks and their relative intensities were then extracted from the time-integrated mass spectrum using Decon2LS (https://omics.pnl.gov/software/decontools-decon2ls), and peaks representing ^13^C compounds were removed. Peaks from the solvent and SOA samples were then aligned with a tolerance of 0.0005 *m*/*z*, and peaks with equal or greater intensity in the solvent than in the samples were also removed. The resultant mass spectra were assigned within a tolerance of 0.0005 *m*/*z* to a formula of [C_c_H_h_O_*x*_N_0–3_ + Na]^+^ and [C_c_H_h_O_*x*_N_0–3_ + H]^+^ for positive ion mode and [C_c_H_h_O_*x*_N_0–3_ – H]^−^ for negative ion mode. The internal calibration of the *m*/*z* axis in both ion modes was verified using the assigned peaks, and the calibration was adjusted where necessary. This internal calibration improved the *m*/*z* accuracy, leading to a few additional assignments for peaks that could not be assigned within 0.0005 *m*/*z* in the uncalibrated mass spectra. Neutral formulas were calculated from the assigned ions by taking into account the ionization mode, and the two modes were clustered together. The mass spectra and elemental ratios presented here refer to the combined peak abundance in the positive and negative ion mode data referenced to formulas of the un-ionized SOA compounds.

### GECKO-A Model

We have conducted modeling of gas-phase chemistry and subsequent SOA formation using the Generator for Explicit Chemistry and Kinetics of Organics in the Atmosphere (GECKO-A) to perform an in-depth investigation of SOA functionality in particles.^44,45^ GECKO-A is a detailed generator of gas-phase chemical mechanisms based on experimental data and observed structure–activity relationships (SAR).^45^ It is coupled to a 0-D photochemical box model enabling the simulation of chamber experiments by treating gas-particle partitioning. Partitioning of oxidation products into the particle phase is treated based on their vapor pressures as estimated using the Nannoolal et al. approach.^46^ GECKO-A does not account for gas-phase chemistry of species with vapor pressure below 10^–13^ atm, which are assumed to be nonvolatile in the condensed phase. The generator accounts for peroxyacyl (RC(O)O_2_) and peroxy (RO_2_) chemistry including reactions with NO_*x*_, HO_*x*_, and RO_2_. For the RO_2_ + HO_2_ reaction, the generator assigns a branching ratio of 80% to hydroperoxide formation and 20% to the creation of an alkoxy radical and the regeneration of another OH. GECKO-A is enriched with SAR estimations of alkoxy radical (RO) decomposition and H-migration reaction rates.^47^ The HO_2_ chemical section also accounts for alkylperoxy radical reactions leading to the formation of hydroperoxides. Note that GECKO-A treats neither autoxidation, dimerization of RO_2_, nor particle-phase chemistry. Chemistry of peroxyacyl nitrates (PANs) is based on the SAR rules by Jenkin et al., covering the reactions of organic peroxy radicals.^48^ There is a limited coverage of specific PAN reactions with −NO_2_ and −HO_2_, and therefore the species follow the SARs reactions pathways of other peroxy radicals. As GECKO-A chemical generator is not able to redact the chemical mechanisms of compounds with more than two double bonds, we were unable to perform an analysis on the functionality distributions of carbonyl groups in SOA produced by naphthalene.

GECKO-A and its photochemical box model have successfully simulated SOA formation by α-pinene photooxidation and dark ozonolysis.^49,50^ In this study, we used GECKO-A photochemical box model to simulate α-pinene photooxidation under low and high NO_*x*_ conditions to track the evolution in SOA functionality with the aim of establishing the dominant chemical routes occurring in our chamber experiments. The initial α-pinene mixing ratio was set to 500 ppb. The low NO_*x*_ scenario includes a steady-state concentration of 1 ppb of NO_*x*_ initially introduced as NO, representative of the background NO_*x*_ mixing ratio in the chamber. NO_*x*_ mixing ratios were fixed to 700 ppb for high NO_*x*_ conditions. The OH concentration was fixed at 1.4 × 10^6^ cm^–3^ to match the steady-state concentration calculated for similar experimental setups reproducing VOC photooxidation in the same chamber.^51^ The RH and temperature were fixed at 38% and 294 K, respectively. Photolysis rates were computed for the photon flux of the UV light used to generate OH in the chamber experiments (250 < λ < 650 nm). As GECKO-A does not include particle nucleation, we assumed pre-existing particles to have a radius of 5 nm and an initial concentration of 10^–4^ cm^–3^.^49,50^ The vapor wall loss is also considered with deposition rate constant of 5 × 10^–3^ s^–1^ based on previous experiments using similar chamber reaction volumes.^52,53^ Functional group distributions in the particle phase were estimated based on the ratio between the total number of carbon atoms per molecule per specific functional group (C_G_) and the total number of carbon atoms measured in the particle phase (C_TOT_).^54^

### Radical 2D-VBS

The radical 2D-volatility basis set model (r2D-VBS) has been described in detail in Schervish and Donahue, but a brief discussion is given here.^55,56^ This model simulates gas-phase chemistry through representative peroxy radical (RO_2_) species and ultimately distributes these products into the 2D-VBS based on volatility or effective saturation mass concentration (C*) using semiempirical kernels for each radical termination reaction. Both autoxidation and dimerization of RO_2_ are included in this model, but due to the simplified chemical kinetics and grouping of RO_2_ species, specific functional groups on closed-shell products cannot be determined. However, autoxidation has been well-documented to lead to organic hydroperoxide (ROOH) formation. This model has previously been shown to reproduce trends associated with α-pinene oxidation under different conditions including addition of NO_*x*_.^55^

For this work, we run the model with an initial input of 500 ppb of α-pinene under both low and high NO_*x*_ scenarios. The low NO_*x*_ scenario includes a steady-state concentration of 1 ppb of NO_*x*_ initially introduced as NO, representative of the background NO_*x*_ mixing ratio in the chamber. In the high NO_*x*_ scenario 700 ppb of NO is introduced and allowed to decay over time. NO_*x*_ cycling was implemented, so most of the NO has been converted to NO_2_ at the end of the simulation. We prescribed an OH concentration of 1.4 × 10^6^ cm^–3^. As this model typically underestimates HO_2_ due to the lack of small carbon-containing molecules that promote conversion of HO_2_ to OH, we prescribed an HO_2_ concentration of 1 × 10^8^ cm^–3^.^56^ We used a wall loss time scale of 1 h for all species. This is to achieve similar product concentrations as seen in the GECKO-A model runs. The time scale used is longer than that used in the GECKO-A simulation as GECKO-A incorporates resuspension of gases from the walls. In addition, the radical 2D-VBS does not include any dynamic gas-particle partitioning, and thus there is no competitive process to wall loss and necessitates wall loss being slower to reach similar concentrations as GECKO-A simulations. To estimate the ROOH concentration in the particle phase, we only included VBS bins which contribute to particles when 200 μg m^–3^ of organic aerosol is present. This leads to 2/3 of the C* = 100 μg m^–3^ bin and 1/6 of the C* = 1000 μg m^–3^ bin being included. All molecules in bins with C* < 100 μg m^–3^ were included, and none were included from bins with C* > 1000 μg m^–3^.

## Results and Discussion

### Environmentally Persistent Free Radicals

Naphthalene SOA showed a significant EPFR signal, while no EPFR signal was observed for α-pinene SOA. This is expected as products of α-pinene oxidation are mostly ring-opened molecules without extensive conjugation and thus do not lead to EPFR formation. Naphthalene is the simplest form of polycyclic aromatic hydrocarbons (PAHs),^57^ which are known to be precursors of EPFR as their oxidation products are expected to retain aromatic or conjugated structures that would stabilize unpaired electrons.^29,58^ The *g* factor of the EPFR signal in this study was around 2.0035 ± 0.0010. A previous study has suggested that *g* factor signals of 2.0035 ± 0.0004 signify carbon-centered (phenoxy) or oxygen-centered (semiquinone) radicals generated from PAH oxidation, so we expect the chemical identity of EPFR to be semiquinone- or phenoxy-type radicals as both radicals are reported to be produced by OH oxidation of naphthalene.^27,58,59^

**Figure 1:**
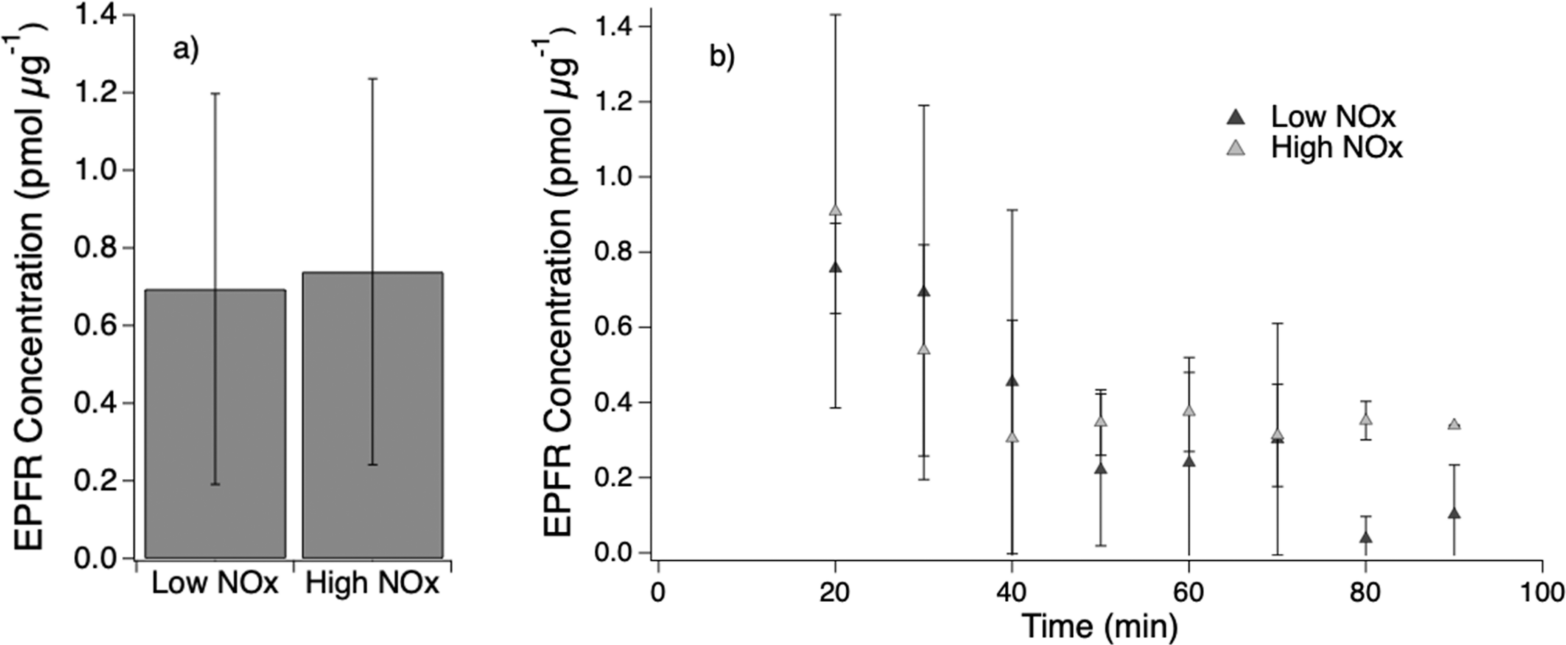
(a) EPFR concentrations in naphthalene SOA generated under low and high NO_*x*_ conditions after 30 min of sample collection. (b) Decay of EPFR in naphthalene SOA generated under low (dark gray) and high NO_*x*_ (light gray) conditions over the span of 80 min after SOA collection.

As shown in Figure 1a, naphthalene SOA generated under both low and high NO_*x*_ conditions exhibited similar concentrations of EPFR with ∼0.7 ± 0.5 pmol (μg SOA)^−1^. The EPFR concentrations were measured for seven SOA samples for each condition. The measurements were conducted after about 20 min of SOA collection completion, and EPFR concentrations were observed to decrease over time followed by flattening, as shown in Figure 1b. EPFR remained present at a lower concentration after 80 min and even after freezing overnight for some samples. While a previous study suggested that carbon-centered radicals are more stable than oxygen-centered radicals,^59^ there was no change in the *g* factor over the course of the decay, indicating no significant changes in radical species. EPFR concentrations were similar between low and high NO_*x*_ environments at each time point after 30 min with e-folding lifetimes of about 42 ± 5 min.

**Figure 2:**
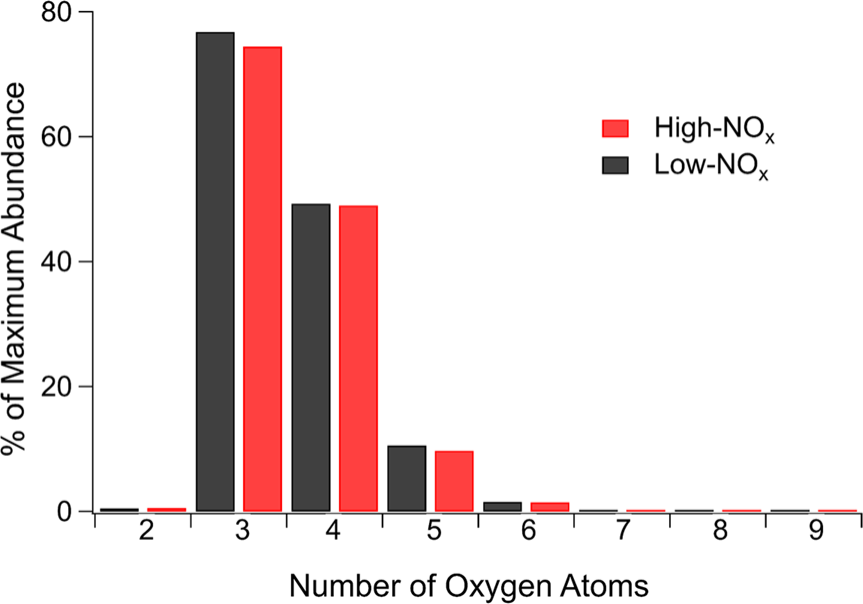
Abundance of C_10_H_6_O_2+_ compounds in naphthalene SOA as determined by high-resolution mass spectrometry.

From the high-resolution mass spectrometry data it is observed that nine of the ten most abundant compounds produced in the chamber under both low and high NO_*x*_ conditions have identical chemical formulas for naphthalene SOA. Results of the high-resolution mass spectrometry data are shown in Table 1 of the HRMS section. The highest six compounds for naphthalene SOA appear in the same order with the same relative intensities (±6%) and exist as ring-retaining structures. Both sets of products display aromatic properties as calculated by their double bond equivalent (DBE) (see further details below). These findings of minimal variation between high and low NO_*x*_ naphthalene SOA composition agree with a previous study.^58^ As such, we would expect that EPFR are produced through the same route and with the same formation efficiency. Figure 2 shows the abundance of C_10_H_6_O_2+_ compounds, respective to their oxygen number (compounds may contain two or more oxygen atoms). C_10_H_6_O_2+_ compounds are assumed to be quinones, which are known precursors to EPFR. There is no discernible effect of NO_*x*_ on the abundance of any oxygen-containing C_10_H_6_O_2+_ compounds. Therefore, in addition to the DBE, an equal amount of C_10_H_6_O_2+_ specifically may account for the comparable EPFR production between low and high NO_*x*_ conditions. Note that naphthalene SOA under a low NO_*x*_ environment produced more ring-retaining compounds such as naphthol, naphthoquinone, and epoxyquinone in the gas phase. Under a high NO_*x*_ environment the major gas-phase products include ring-opening products such as 2-formyl cinnamaldehyde, phthaldialdehyde, and phthalic anhydride.^58^ Further research is required to identify chemical identities of EPFR generated under low and high NO_*x*_ conditions.

**Table 1:** Top Ten Peaks in Each SOA Formation Condition Listed by Relative Peak Abundance, Normalized to the Highest Peak in Each Spectrum, Including Their Neutral Mass and Assigned Neutral Formula

naphthalene low NO_*x*_			naphthalene high NO_*x*_			α-pinene low NO_*x*_			α-pinene high NO_*x*_		
rel abundance (%)	neutral mass (Da)	formula	rel abundance (%)	neutral mass (Da)	formula	rel abundance (%)	neutral mass (Da)	formula	rel abundance (%)	neutral mass (Da)	formula
100	134	C_8_H_6_O_2_	100	134	C_8_H_6_O_2_	100	182	C_10_H_14_O_3_	100	166	C_10_H_14_O_2_
86	162	C_9_H_6_O_3_	81	162	C_9_H_6_O_3_	95	198	C_10_H_14_O_4_	93	172	C_8_H_12_O_4_
79	192	C_10_H_8_O_4_	74	174	C_10_H_6_O_3_	82	172	C_8_H_12_O_4_	88	198	C_10_H_14_O_4_
77	174	C_10_H_6_O_3_	71	176	C_10_H_8_O_3_	81	166	C_10_H_14_O_2_	85	138	C_8_H_10_O_2_
57	176	C_10_H_8_O_3_	62	192	C_10_H_8_O_4_	74	138	C_8_H_10_O_2_	81	182	C_10_H_14_O_3_
56	166	C_8_H_6_O_4_	62	166	C_8_H_6_O_4_	70	126	C_7_H_10_O_2_	77	126	C_7_H_10_O_2_
55	148	C_9_H_8_O_2_	60	189	C_10_H_7_O_3_N	66	152	C_9_H_12_O_2_	75	168	C_9_H_12_O_3_
49	190	C_10_H_6_O_4_	49	190	C_10_H_6_O_4_	63	164	C_10_H_12_O_2_	70	186	C_9_H_14_O_4_
48	208	C_10_H_8_O_5_	48	150	C_8_H_6_O_3_	62	154	C_8_H_10_O_3_	69	124	C_8_H_12_O_1_
40	150	C_8_H_6_O_3_	46	148	C_9_H_8_O_2_	60	140	C_7_H_8_O_3_	67	122	C_8_H_10_O_1_

### Reactive Oxygen Species

**Figure 3:**
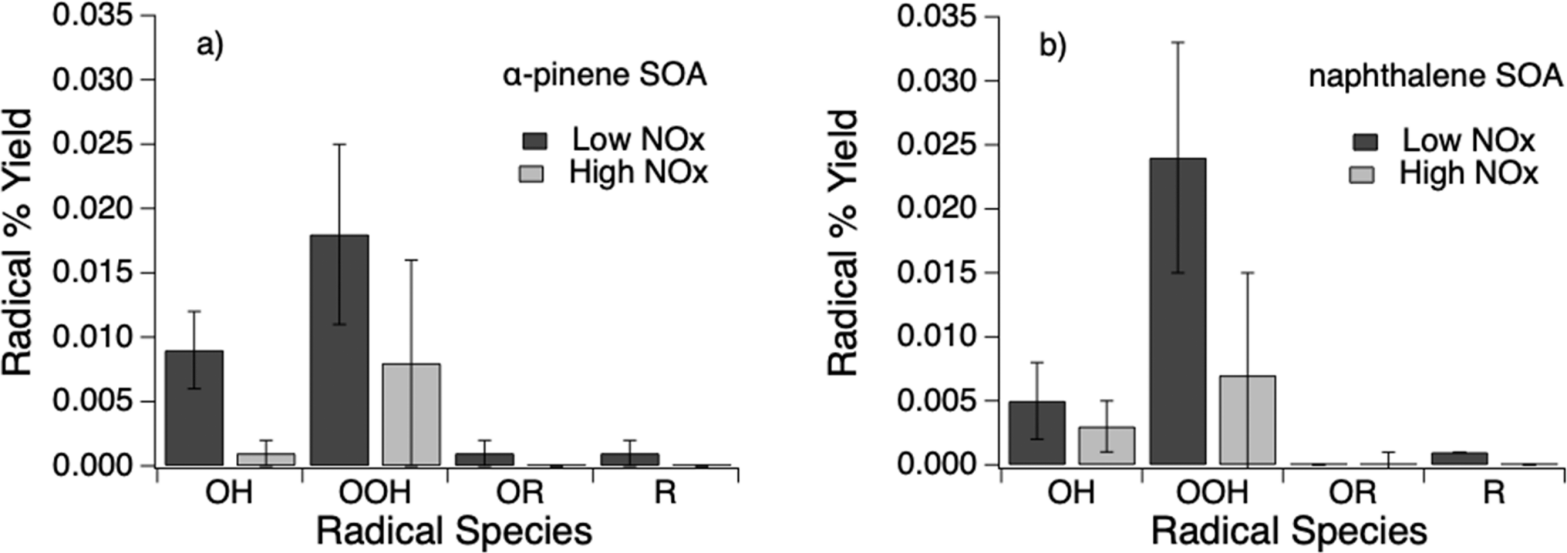
Molar yields of BMPO radical adducts (BMPO-OH, BMPO-OOH, BMPO-OR, and BMPO-R) in water extracts of (a) α-pinene and (b) naphthalene SOA generated under low and high NO_*x*_ conditions.

Figure 3a shows that α-pinene SOA generated under the low NO_*x*_ condition mainly generates OH radicals and superoxide, which is consistent with our previous study.^21^ OH radicals can be generated by the decomposition of ROOH, and superoxide can be formed by OH oxidation of primary and secondary alcohols and unimolecular decomposition of α-hydroxyperoxy radicals.^21,22^ In Figure 3b, naphthalene SOA generated under the low NO_*x*_ condition shows the dominant superoxide formation, which is most likely generated via redox reactions of quinones.^28,29^ The formation of organic radicals are relatively minor for both SOA.

For both naphthalene and α-pinene SOA generated under high NO_*x*_ conditions, ROS production was diminished. For α-pinene SOA, the total radical yield (the percentage of moles of BMPO radical adducts to the total moles of SOA) decreased from ∼0.029% to ∼0.009%, with OH and superoxide formation decreased by a factor of ∼9 and ∼2, respectively. For naphthalene SOA, the total radical yield was reduced from ∼0.030% to ∼0.010%, with OH and superoxide reduction by a factor of ∼1.5 and ∼3.5, respectively. SOA extracts were slightly more acidic for high NO_*x*_ SOA compared to low NO_*x*_ SOA, consistent with Kautzman et al., who found an enhancement of acid formation for naphthalene SOA produced in a high NO_*x*_ environment.^58^ Note that our recent study showed α-pinene and naphthalene SOA generated under low NO_*x*_ would have higher ROS production at lower pH,^60^ but the influence of NO_*x*_ has a greater effect on ROS yield than the pH of the system. A previous study has indicated that acid may reduce the formation of semiquinones,^27^ which may contribute to the reduction of ROS formation.

**Figure 4:**
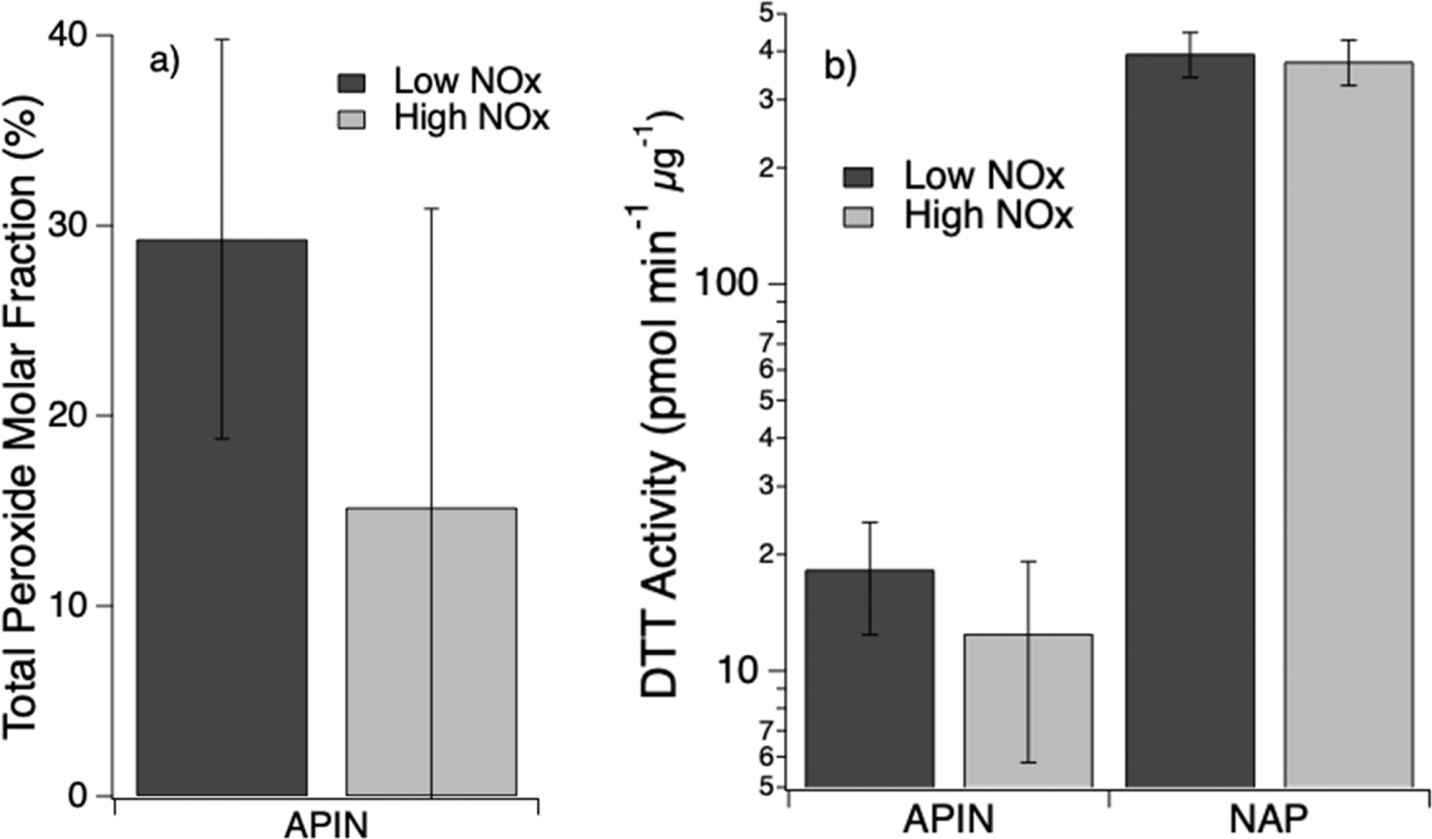
(a) Total peroxide concentrations and (b) DTT activities of α-pinene and naphthalene SOA produced under low and high NO_*x*_ conditions. Error bars represent the standard deviation.

Given ROOH is an important source of ROS, we have quantified peroxides using an iodometric spectrophotometric method. As shown in Figure 4a, α-pinene SOA generated under the low NO_*x*_ conditions contained substantial amounts of peroxides (∼29 ± 11%), which decreased significantly by a factor of ∼2 for SOA generated under high NO_*x*_ conditions. There was not a detectable amount of peroxide for naphthalene SOA. Given that organic peroxides are reported to contribute to oxidative potential,^61^ we performed the DTT assay on both samples of naphthalene and α-pinene SOA, as shown in Figure 4b. Naphthalene SOA has a significantly higher DTT activity by a factor of 4 than α-pinene SOA, most likely due to redox activity of quinones contained in naphthalene SOA.^62^ The oxidative potential is slightly higher for α-pinene SOA formed under low NO_*x*_ compared to those under high NO_*x*_, while there is no significant difference for naphthalene SOA. It has been shown that DTT activity of α-pinene SOA is insensitive to NO_*x*_,^63^ while NO_*x*_ was reported to enhance oxidative potential of isoprene SOA.^61^

### SOA Modeling

For α-pinene oxidation, the fate and reaction pathways of peroxy radicals (RO_2_) are critical for SOA formation. There are competitions of NO_*x*_ and HO_2_ to react with RO_2_ radicals leading to different products as follows:
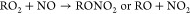
R1
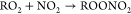
R2
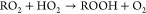
R3Note that (R2) is only relevant for acylperoxy radicals (R–C(O)OO), and it is an equilibrium process. In addition, NO_*x*_ can decrease the abundance of ROOH by suppressing autoxidation and the formation of HOM, which can be significant source of ROS.^64^

**Figure 5:**
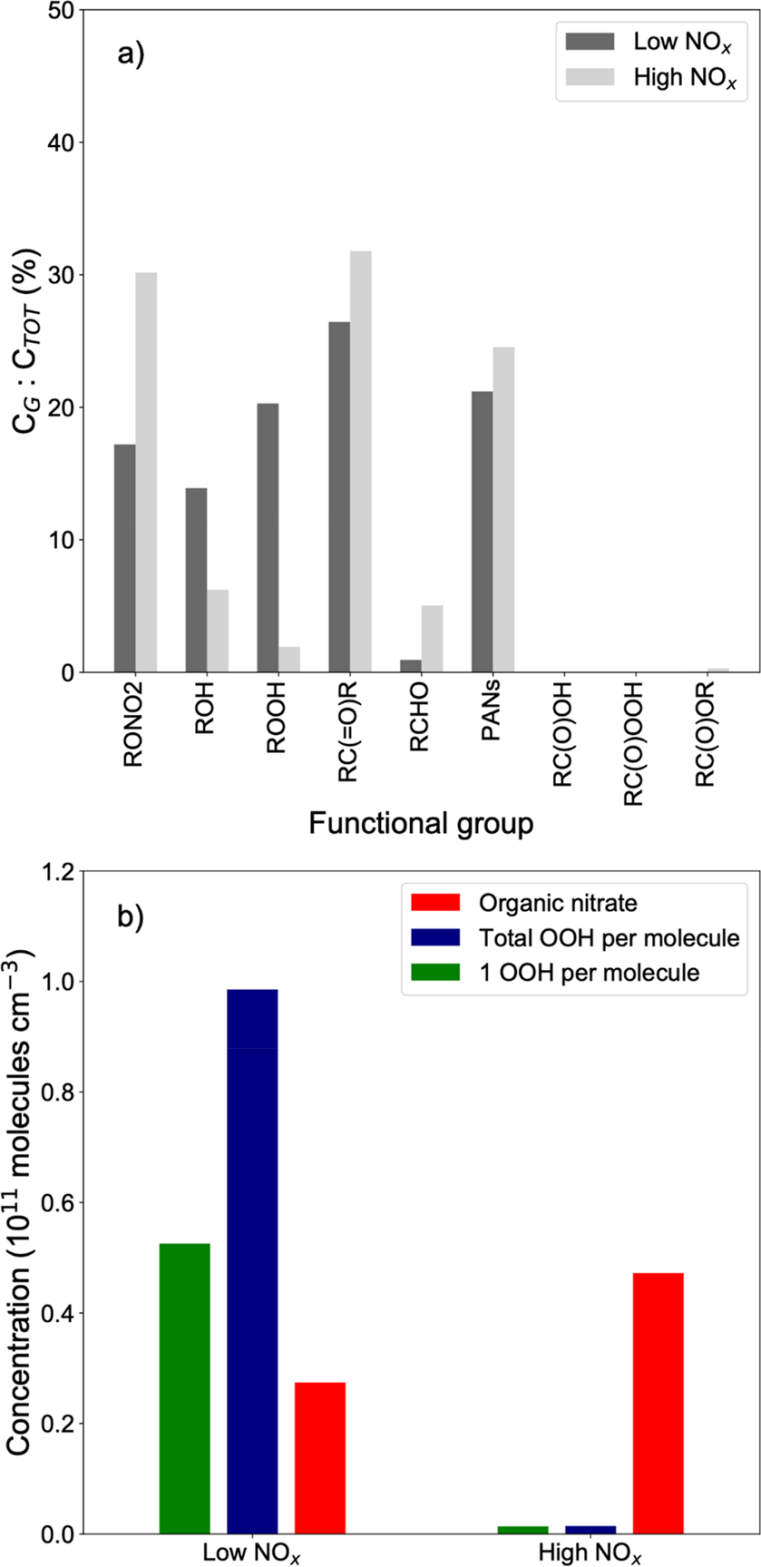
(a) Functional group distributions in α-pinene SOA simulated by the GECKO-A box model for the photooxidation of α-pinene under low and high NO_*x*_ conditions. (b) Modeled concentrations of organic hydroperoxides and organic nitrates from α-pinene oxidation under low and high NO_*x*_ conditions by r2D-VBS. The green bars represent OOH formation with an allowed production of one peroxide per molecule, whereas the blue bars represent OOH formation with OOH production at each step of autoxidation. The red bar represents the organic nitrate formation in the presence of NO_*x*_.

Figure 5a shows the SOA functional group distributions for simulated α-pinene photooxidation SOA under low and high NO_*x*_ conditions by GECKO-A. Under low NO_*x*_, RO_2_ would mainly react with HO_2_ to form ROOH, accounting for 20% of carbon atoms, which is comparable to the measured peroxide yield of ∼29% (Figure 5a). 13% of carbon atoms are associated with alcohols (ROH). Under high NO_*x*_ conditions, RO_2_ are terminated by NO_*x*_ forming organic nitrates (RONO_2_),^40^ and ROOH is expected to be very low. It is estimated that around 30% of carbon atoms would be associated with RONO_2_, in addition to around 25% associated with PANs. Note that PAN potentially decompose to additional peroxy radicals,^65,66^ but this process is not treated in the model, which may lead to less alcohol predicted in the model than is observed experimentally. Slightly higher formation of ketone (RCOR) and aldehyde (RCHO) species is predicted under high NO_*x*_ environments.

As GECKO-A does not account for autoxidation, we applied the radical 2D-VBS model which considers autoxidation. Because specific functional group information about the oxidized SOA is unknown, we made assumptions about the −OOH groups present on the molecules. In the first case, it was assumed that every molecule that has undergone autoxidation has only one −OOH group that can decompose and lead to ROS formation (one OOH per molecule). In the second case, we counted every step of autoxidation a molecule has undergone as being an additional −OOH group that can decompose (total OOH per molecule). In both cases products from the RO_2_ + HO_2_ reaction were included regardless of degree of autoxidation (but were counted as an additional −OOH group in case 2). We included these cases as some ROOH may decompose prior to analysis, a process that is not accounted for in the model. While this model treats dimerization, it is unclear to what extent dimers may decompose or hydrolyze in aerosol; therefore, we only included their contribution to the total −OOH functionality if they underwent any autoxidation.

Figure 5b shows a clear reduction in the −OOH groups as a result of the addition of NO_*x*_, consistent with the results from GECKO-A. The percentage reduction in each case is between 95 and 98%. While the mechanism for the production of these hydroperoxides is different in these two models, the excellent agreement for the relative reduction due to the addition of NO_*x*_ to the system provides evidence of this phenomenon occurring whether autoxidation is taking place and RO_2_ are primarily terminating with each other or if RO_2_ is primarily reacting with HO_2_. In reality, both of these termination reactions are occurring to different extents under different conditions depending on NO:HO_2_, but both are clearly repressed with the addition of NO_*x*_.

Figure 5b also shows the total organic nitrates formed in both scenarios. While termination with NO_*x*_ is competitive under both scenarios, when NO_*x*_ is present in small amounts, it does not totally dominate over autoxidation, allowing for the formation of some organic nitrates that have −OOH functionality due to prior autoxidation. However, under high NO_*x*_ conditions, the RO_2_ + NO dominates autoxidation, leading to a marked decrease in hydroperoxide functionality. The organic nitrates alone do not totally compensate for the decrease in −OOH functionality because formation of organic nitrates from the reaction of RO_2_ + NO only accounts for 25% of the yield of this reaction in the model. However, as these species tend to be lower in volatility than the products formed from the alkoxy radical pathway within this model framework, the particle-phase organic nitrates account for much more than 25% of the particle phase yields. The increase in particle-phase RONO_2_ species by roughly a factor of 2 from the low to high NO_*x*_ scenarios is also consistent with the results from GECKO-A.

### High-Resolution Mass Spectrometry

**Figure 6:**
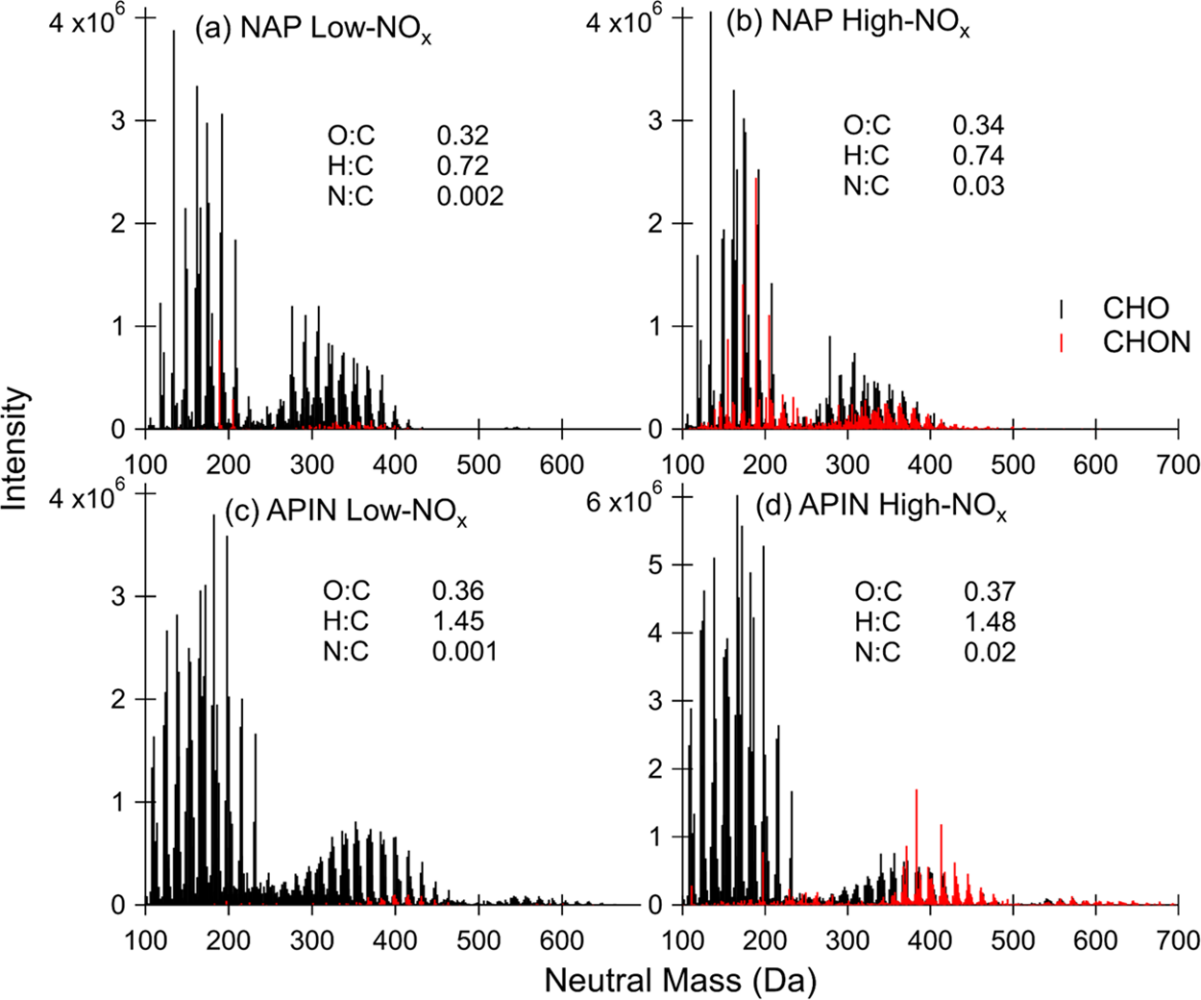
High-resolution mass spectra and intensity-normalized average elemental ratios for (a) naphthalene (NAP) low NO_*x*_ SOA, (b) NAP high NO_*x*_ SOA, (c) α-pinene (APIN) low NO_*x*_ SOA, and (d) APIN high NO_*x*_ SOA. Compounds containing only carbon, hydrogen, and oxygen (CHO) are shown in black while compounds containing carbon, hydrogen, oxygen, and nitrogen (CHON) are shown in red.

We observed that both naphthalene and α-pinene SOA show significant incorporation of nitrogen under high NO_*x*_ conditions as predicted by the GECKO-A and r2D-VBS model. As shown in Figure 6, the addition of NO_*x*_ during SOA formation leads to the production of many nitrogen-containing compounds. There are also low abundances of nitrogen-containing compounds (CHON) formed under low NO_*x*_ conditions (Figure 6a,c) as background levels of NO_*x*_ in the smog chamber were around 1 ppb. While our mass spectrometry method does not provide individual functional group information, based on previous work, we expect the nitrogen-containing groups in the naphthalene SOA to be mostly nitroaromatics and in the α-pinene SOA to be organic nitrates (RONO_2_).^67,68^ SOA composition agrees relatively well with previous work on α-pinene and naphthalene high and low NO_*x*_ SOA.^69−72^ Both SOA types have distinct monomer (approximately <250 Da) and dimer (250–500 Da) regions. The α-pinene SOA also has a small trimer region above 500 Da. The major peaks in both naphthalene SOA conditions generally have formulas of C_8–10_H_6–8_O_2–5_ (see Table 1 for the top 10 most abundant peaks in each spectrum). The major peaks in both α-pinene SOA conditions have formulas of C_8–10_H_12–16_O_2–5_. For the α-pinene SOA, there are also a few C_7_ compounds that are abundant and less typical of previously reported α-pinene SOA. These C_7_ compounds may be a result of in-source fragmentation due to the spray voltage being too high during mass spectrometry analysis. They are not evident in the aromatic naphthalene SOA, which should be less susceptible to fragmentation.

Table 1 shows the top ten products for naphthalene and α-pinene SOA under high and low NO_*x*_ conditions. Nine of the ten top naphthalene SOA products are shared for the high and low NO_*x*_ environments. The two deviations for naphthalene SOA were C_10_H_8_O_5_ and C_10_H_7_O_3_N. The naphthalene high NO_*x*_ environment saw the incorporation of nitrogen into the final product, forming a nitroaromatic. Only the top five out of ten α-pinene SOA products were the same for high and low NO_*x*_ conditions. None of the remaining five products of α-pinene SOA in the high NO_*x*_ environment included organic nitrates. In the complete product list for α-pinene SOA, organic nitrate compounds were present, agreeing with the aforementioned studies that show the formation of these compounds in a high NO_*x*_ environment. Also, there may be fewer nitrogen-containing compounds measured by HRMS in the α-pinene SOA because some of the organic nitrates may have hydrolyzed in water before they were able to be analyzed; for instance, tertiary nitrates derived from isoprene have been estimated to have hydrolysis lifetimes of <10 s when dissolved in water.^73^

**Figure 7:**
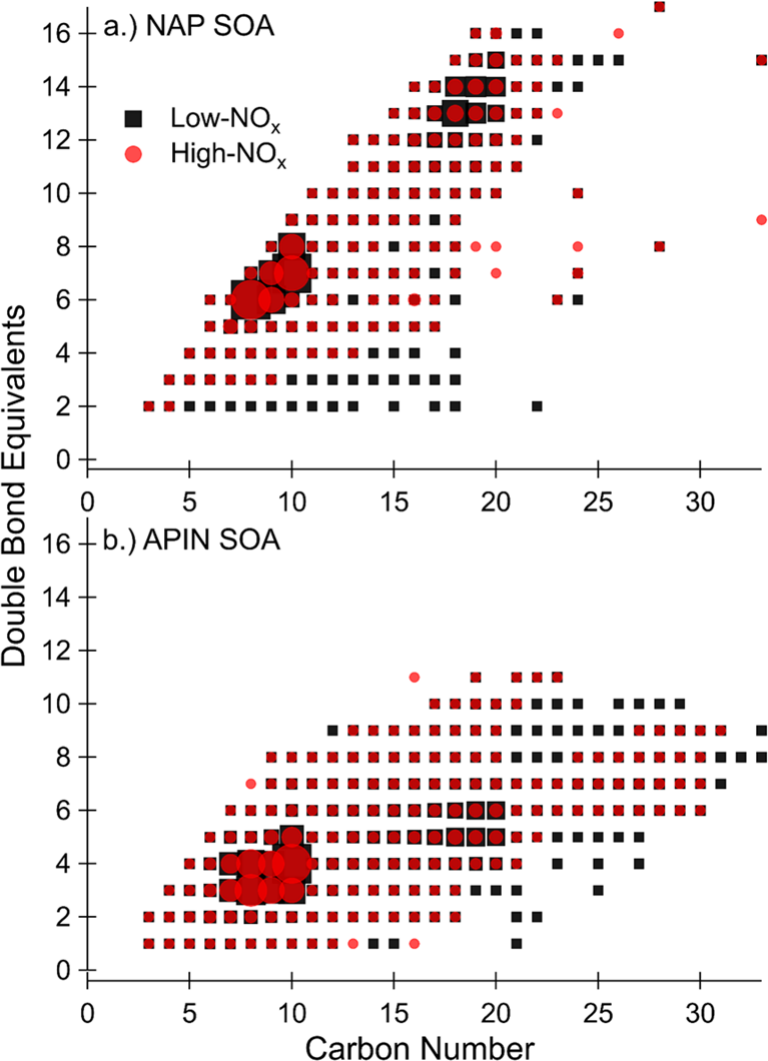
Double-bond equivalents as a function of carbon number for (a) naphthalene SOA and (b) α-pinene. SOA formed under high NO_*x*_ conditions, excluding nitrogen-containing compounds, is shown in red circles, and SOA formed under low NO_*x*_ conditions is shown in black squares. Marker size is scaled to the relative summed intensity at each point.

To look for evidence of changes in the reaction pathways described in chemical reactions R1–R3, specifically a reduction in peroxide functional group formation with the addition of NO_*x*_, we examined the double-bond equivalents (DBE) as a function of carbon number for the compounds that do not contain nitrogen, shown in Figure 7. Equation R3 predicts the formation of peroxide functional groups which have a DBE of 0. Under high NO_*x*_ conditions, we expect (R1) to compete with (R3), leading to a carbonyl group with a DBE of 1. Therefore, if the addition of NO_*x*_ reduces the formation of peroxides, the DBE distribution of the CHO compounds should show fewer low-DBE compounds under high NO_*x*_ conditions. Looking at Figure 7a, this seems to be the case for naphthalene SOA. Some of the CHO compounds with low DBE which form under low NO_*x*_ conditions are not formed under high NO_*x*_ conditions. This pattern appears less evident for the α-pinene conditions, shown in Figure 7b, although there are some low-DBE peaks present under low NO_*x*_ conditions which are not present under the high NO_*x*_ conditions. The abundance of peroxide functionalities determined by the ROS measurements are also more similar between the low and high NO_*x*_ conditions for α-pinene than for the naphthalene conditions, so our DBE comparison may not be sensitive enough to observe a shift for α-pinene SOA. Finally, the higher aromaticity of naphthalene, which is shown by higher DBE in Figure 7a, corroborates the EPFR results, as aromaticity is required for EPFR.^59^

## Discussion

The experiments were conducted using high mixing ratios of α-pinene and NO_*x*_ to produce enough material for the EPR and HRMS analysis. This limitation does not apply to the models, which can assume any starting conditions. By maintaining the same temperature, RH, and total absorbing organic aerosol mass, but with an α-pinene and NO_*x*_ mixing ratio reduced to 200 ppt and 50 ppb, respectively, r2D-VBS predicts a reduction of −OOH functionality by a factor of 25 from the NO_*x*_-free condition. If we represent more pristine conditions with 50 ppt of NO_*x*_, the reduction of −OOH functionality is only 20% compared to the NO_*x*_-free condition. These percentages reflect only counting one −OOH group per molecule (case 1) because, as noted previously, the different methods give quite similar relative reductions. The reduction of −OOH functionality may be due to the fact that under 50 ppt NO_*x*_ competitiveness of autoxidation is enhanced and may be dominant over that of a reaction with NO. In the chamber NO reacted quickly to form NO_2_. As there was an initial concentration of 700 ppb NO, it can be assured that enough NO remained in the chamber to make the reaction of RO_2_ with NO dominant over other loss processes. With 1 ppb or greater NO maintained in the chamber and equal amounts of RO_2_, the isomerization of RO_2_ with the first-order rate of ∼0.1 s^–1^ and the reaction of RO_2_ + NO are competitive.^48,74^ Under 50 ppt NO_*x*_ this may no longer be the case, and autoxidation will most likely be the major process, while RO_2_ can still undergo a reaction with NO.

The GECKO-A and 2D-VBS models predict the reduction of hydroperoxides under high NO_*x*_ conditions. The formation of organic nitrates in the presence of NO_*x*_ was predicted in both models, which can be interpreted as a competitive alternative pathway to hydroperoxide formation for the RO_2_ termination. The presence of organic nitrates under high NO_*x*_ conditions was also confirmed experimentally using high-resolution mass spectrometry. The model outcomes, however, predicted a higher reduction by 92–98% in hydroperoxides than was observed experimentally in the reduction of O_2_^–^ and HO_2_ by 25–75% and the reduction of OH by 70–90% for α-pinene SOA.

Because these models are both gas-phase models, the apparent discrepancy may be due to chemistry in the particle phase. In addition, particle-phase processes associated with nonideal mixing and particle phase state are not explicitly considered in these models. When particles adopt amorphous semisolid or glassy states, kinetic limitations of bulk diffusion can retard mass accommodation, heterogeneous reactions, and partitioning.^75−77^ This may contribute to the potential overrepresentation of multifunctionalized NO_2_ and OH bearing compounds in the presence of NO_*x*_ which can lead to production of more volatile compounds.^14^ Moreover, the occurrence of liquid–liquid phase separation can also significantly impact SOA partitioning.^78^ These aspects may warrant further investigations in future studies.

While organic hydroperoxides are regarded as a major ROS source, it is unclear if peroxyacetyl nitrates and other highly functionalized nitrates might contribute to ROS formation. Some of these compounds might decompose and through a series of subsequent reactions form the peroxy radical, contributing to the production of ROS.^65^ Note that the GECKO-A model accounts for the decomposition of PAN in the gas phase; given there is little variation between GECKO-A and 2D-VBS, we do not expect this process to be a major process contributing to ROS formation.

Our results are in contrast with the findings from Chowdhury et al., where there was more ROS production from aged naphthalene and α-pinene SOA in the presence of NO_*x*_. Note that our study agrees with their finding that total peroxide production from aged α-pinene SOA decreases in the presence of NO_*x*_.^28^ The discrepancy between these studies may be due to methodology. SOA in the Chowdhury study was generated in an oxidative flow reactor, with an aging period equivalent to 3 days in the atmosphere. Concentrations of naphthalene and α-pinene as well as NO_*x*_ were on the same order of magnitude, but NO_*x*_ was added as 2% NO_2_. Further studies are warranted to investigate ROS formation by SOA as a function of chemical aging time.

## Conclusions

In this study we measured EPFR and ROS associated with naphthalene and α-pinene SOA generated under low and high NO_*x*_ conditions. We found that α-pinene SOA does not contain significant amounts of EPFR, while naphthalene SOA contains EPFR with a particle-mass-normalized concentration of ∼0.7 ± 0.5 pmol μg^–1^. EPR measurements show that NO_*x*_ exhibits minimum impacts on EPFR production, and high-resolution mass spectrometry measurements indicate that there is also no discernible effect of NO_*x*_ on the abundance of C_10_H_6_O_2+_ compounds, which are likely to be quinones which would produce EPFR. α-Pinene and naphthalene SOA generated under low NO_*x*_ conditions mainly produce OH radicals and superoxide in the aqueous phase, which is suppressed substantially for SOA generated in the presence of NO_*x*_; the total radical yield decreased from 0.029% to 0.009% for α-pinene SOA and from 0.030% to 0.010% and for naphthalene SOA.

The reduction of ROS formation for SOA generated in the presence of NO_*x*_ is due to suppression of the formation of organic hydroperoxides as indicated by peroxide measurements and the reduction of total oxidative potential as measured by the DTT assay. The GECKO-A modeling finds that RO_2_ is terminated by NO_*x*_ forming organic nitrates (RONO_2_), and ROOH is expected to be very low under high NO_*x*_ conditions. Further modeling by r2D-VBS, which considers autoxidation for the formation of highly oxygenated organic molecules with multiple −OOH groups, also shows a clear reduction in ROOH upon NO_*x*_ addition. High-resolution mass spectrometry confirmed the production of numerous nitrogen-containing compounds. In addition, some of the compounds with low double-bond equivalents, which is likely to be correlated to ROOH formation, form in higher concentration under low NO_*x*_ conditions than under high NO_*x*_ conditions. In conclusion, our experimental and modeling results clearly demonstrate that NO_*x*_ alters the fate and reaction pathways of peroxy radicals and hence SOA chemical composition, resulting in the reduction of ROS formation by SOA generated under high NO_*x*_ conditions. The results from this study of aqueous extracts of naphthalene and α-pinene SOA provide useful insights into aqueous-phase processing of organic aerosols in the atmosphere as well as ROS formation and oxidative stress upon inhalation and respiratory deposition of SOA into lung lining fluid.
